# *N*-Phenyl-[1,1′-biphen­yl]-2-carboxamide

**DOI:** 10.1107/S2414314626005754

**Published:** 2026-06-05

**Authors:** Nour El Houda Guerah, Attia Tarek, Allaoui Mahfoud Mounib, Jean-Claude Daran, Eric Manoury

**Affiliations:** a Laboratoire des sciences analytiques materiaux et environnement (LSAME) Université Oum El Bouaghi Oum El Bouaghi 04000 Algeria; b Département des Sciences de la Matière, Université d’Oum El Bouaghi, 04000, Algeria; c Laboratoire de Chimie de Coordination, UPR-CNRS 8241 205 route de Narbonne 31077 Toulouse cedex France; Katholieke Universiteit Leuven, Belgium

**Keywords:** crystal structure, carboxamide, biphenyl derivative, hydrogen bonding, C—H⋯π inter­actions

## Abstract

The title mol­ecule, C_19_H_15_NO, contains a carboxamide fragment in which the amide N atom is bonded to a phenyl group, while the carbonyl C atom is attached to a biphenyl unit. In the crystal, mol­ecules are linked by N—H⋯O hydrogen bonds, forming chains running parallel to the *a* axis. These chains are further connected by C—H⋯π inter­actions, resulting in a three-dimensional supra­molecular network.

## Abstract 2

The title mol­ecule contains a carboxamide fragment in which the amide N atom is bonded to a phenyl group, while the carbonyl C atom is attached to a biphenyl unit. In the crystal, mol­ecules are linked by N—H⋯O hydrogen bonds, forming chains running parallel to the a axis. These chains are further connected by C—H⋯π inter­actions, resulting in a three-dimensional supra­molecular network.

## Graphical abstract

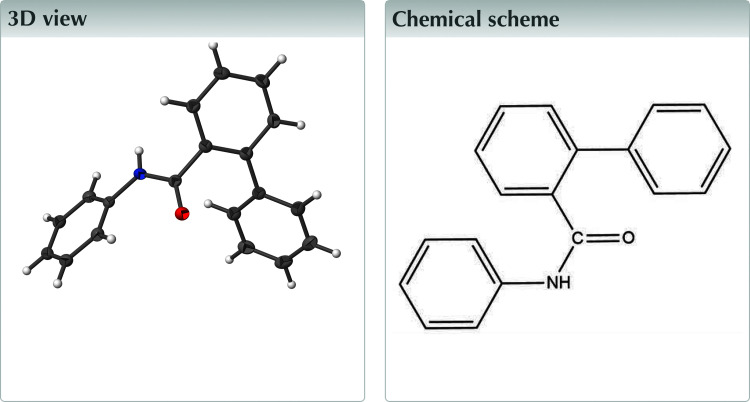

## Structure description

Biphenyl derivatives represent an important class of aromatic compounds owing to their conformational flexibility and structural diversity (Jain *et al.*, 2017 ▸; Landeros-Rivera & Hernańdez-Trujillo, 2022 ▸). Functionalized biphenyl systems bearing carb­oxy­lic acid or amide groups have attracted considerable inter­est for their structural and coordination properties (Sienkiewicz-Gromiuk *et al.*, 2014 ▸; Wang *et al.*, 2004 ▸; Yu *et al.*, 2006 ▸), as well as for their biological relevance (Sharma *et al.*, 2010 ▸; van ’t Hof *et al.*, 2004 ▸; Mukherjee *et al.*, 2016 ▸; Zhao *et al.*, 2017 ▸). The amide functional group is well known for its strong hydrogen-bonding ability and its role in directing supra­molecular organization in the solid state. The combination of a biphenyl scaffold with an amide linkage provides a versatile structural platform capable of promoting inter­molecular hydrogen bonding and π–π stacking inter­actions, which are key factors in supra­molecular self-assembly processes (Gao *et al.*, 2022 ▸; Yao *et al.*, 2025 ▸). Recent crystallographic studies of substituted biphenyl derivatives further highlight the influence of these inter­actions on mol­ecular conformation and crystal packing (Nodera *et al.*, 2025 ▸). In this context, we report herein the synthesis and crystal structure of the title comnpound, C_19_H_15_NO.

**Figure 1:**
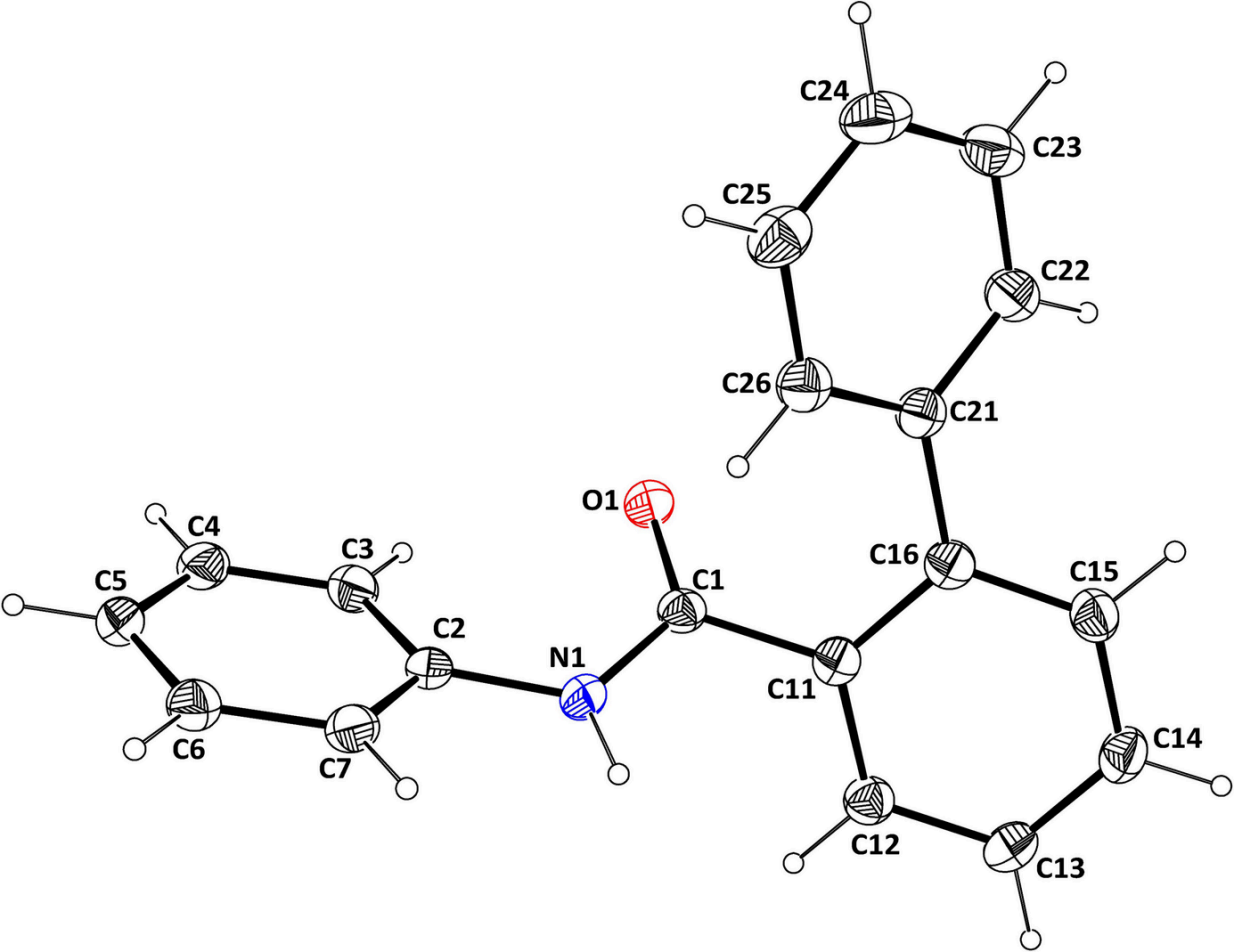
Mol­ecular structure of the title compound with the labelling scheme. Displacement ellipsoids are drawn at the 50% probability level. H atoms are shown as small spheres of arbitrary radius.

The title compound **1** crystallizes in the triclinic space group *P*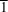 with one mol­ecule in the asymmetric unit (Fig. 1 ▸). The mol­ecular structure consists of a carboxamide fragment, C—N(H)—C(=O)—C, in which the amide N atom is bonded to a phenyl group and the carbonyl C atom is bonded to a biphenyl unit. The C—N, N—C and C=O bond lengths are in agreement with those observed in related compounds (see below). The amide fragment C—N(H)—C(=O)—C is essentially planar, with the largest deviation from the mean plane being 0.0413 (5) Å for atom C2. The phenyl ring C2—C7 is twisted by 24.70 (4)° with respect to the amide mean plane, while the phenyl ring attached to the carbonyl group is inclined by 55.67 (4)°. The phenyl and biphenyl groups are in the *trans* position with respect to the C1—N1 bond. The dihedral angle between the biphenyl rings is 40.67 (6)°.

**Figure 2:**
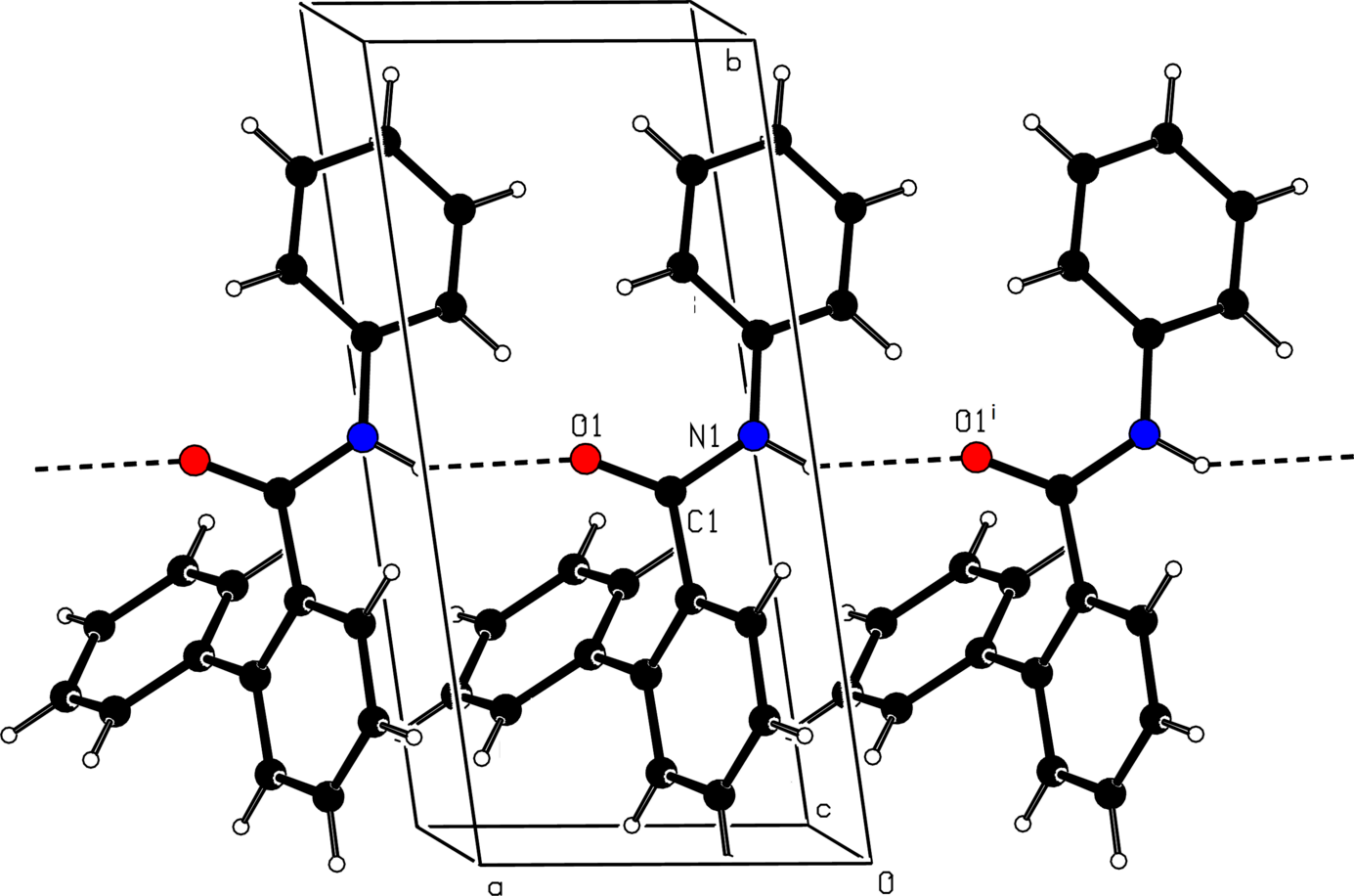
Partial packing view showing the formation of N—H⋯O hydrogen-bonded chains running parallel to the *a* axis. Symmetry code as in Table 1 ▸.

In the crystal, mol­ecules are linked by N1—H1⋯O1 hydrogen bonds, generating chains running parallel to the *a* axis (Table 1 ▸, Fig. 2 ▸). A weak C7—H7⋯O1 contact also contributes to the crystal packing. In addition, weak C—H⋯π inter­actions (Table 1 ▸) involving atoms C13 and C24 and the centroid of the C2—C7 phenyl ring further consolidate the packing. These inter­actions connect the N—H⋯O hydrogen-bonded chains into a three-dimensional supra­molecular network.

A search of the Cambridge Structural Database (CSD, version 5.36; Groom *et al.*, 2016 ▸), based on the Ph—NH—C(=O)—C(*R*) fragment, revealed ten related structures containing a phenyl group attached to the amide N atom and different substituents attached to the carbonyl C atom. A comparison of selected bond lengths and angles is given in Table 2 ▸.

## Synthesis and crystallization

**Figure 3:**
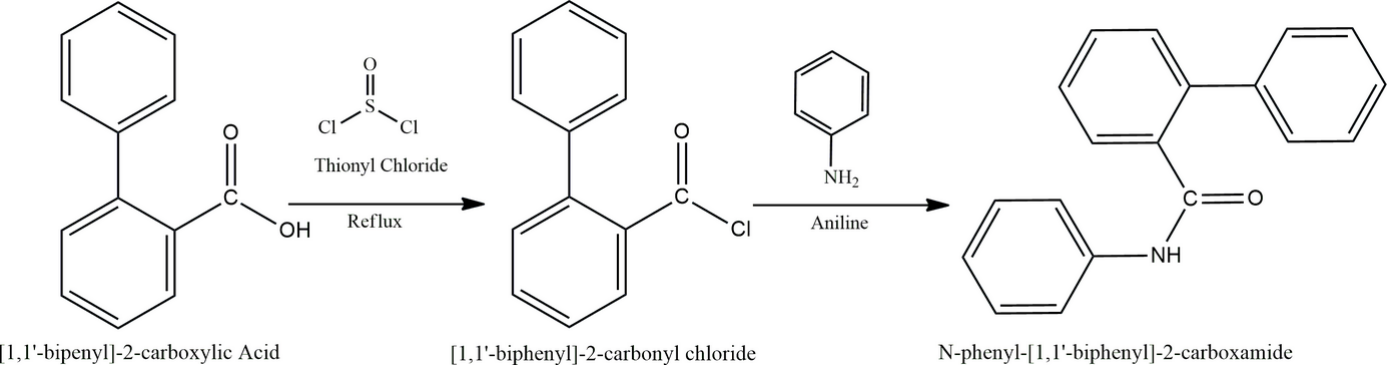
Synthesis of *N*-phenyl-[1,1′-biphen­yl]-2-carboxamide.

2-Bi­phenyl­carb­oxy­lic acid (1.63 g, 10 mmol) was dissolved in toluene (50 ml) and treated dropwise with thionyl chloride SOCl_2_ (1.67 ml, 15 mmol) under stirring at 323–333 K. The reaction mixture was maintained at this temperature to allow formation of the corresponding acyl chloride. Aniline (1.03 g, 10 mmol) was then added dropwise, and the mixture was heated under reflux for 3 h. After completion of the reaction, the solvent was removed under reduced pressure. The crude solid was purified by recrystallization from ethanol solution to afford the title amide as a white solid (yield = 80%). Crystals suitable for single-crystal X-ray diffraction were obtained by slow evaporation of a solution of the compound in ethanol at room temperature. The reaction scheme is shown in Fig. 3 ▸.

## Refinement

Crystal data, data collection and structure refinement details are summarized in Table 3 ▸.

## Supplementary Material

Crystal structure: contains datablock(s) shelx, I. DOI: 10.1107/S2414314626005754/vm4077sup1.cif

Structure factors: contains datablock(s) I. DOI: 10.1107/S2414314626005754/vm4077Isup2.hkl

Supporting information file. DOI: 10.1107/S2414314626005754/vm4077Isup3.cml

CCDC reference: 2558356

Additional supporting information:  crystallographic information; 3D view; checkCIF report

## Figures and Tables

**Table 1:** Hydrogen-bond geometry (Å, °) *Cg*1 is the centroid of the C2–C7 ring.

*D*—H⋯*A*	*D*—H	H⋯*A*	*D*⋯*A*	*D*—H⋯*A*
N1—H1⋯O1^i^	0.877 (16)	2.307 (16)	3.0790 (12)	146.9 (13)
C3—H3⋯O1	0.95	2.38	2.9035 (15)	114
C7—H7⋯O1^i^	0.95	2.64	3.2433 (13)	122
C13—H13⋯*Cg*1^ii^	0.95	2.78	3.5815 (13)	142
C24—H24⋯*Cg*1^iii^	0.95	2.94	3.8444 (13)	160

Symmetry codes: (i) 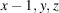; (ii) 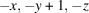; (iii) 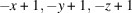.

**Table 2:** Comparison of selected distances and angles (Å, °) in related compounds having a similar C(Ph)—NH—C(O)—C(*R*) fragment

Compound	N—C(O)	N—C(Ph)	C=O	C(O)—C(*R*)	C—N—C	N—C—C
**1**	1.3626 (14)	1.4198 (14)	1.2283 (13)	1.5001 (15)	126.70 (9)	114.07 (9)
CIBPIM	1.332	1.400	1.234	1.508	127.1	114.0
CIBPIM01	1.340	1.421	1.232	1.505	128.2	114.9
LASHEU	1.335	1.431	1.236	1.495	122.1	118.5
MANDIP	1.354	1.409	1.232	1.487	128.4	115.0
MANDIP01	1.350	1.416	1.237	1.497	127.3	115.3
MANDIP02	1.352	1.410	1.233	1.500	127.8	114.3
MANDIP03	1.353	1.412	1.233	1.499	127.9	114.1
NUKVOH	1.355	1.420	1.226	1.595	125.4	115.8
YEGJID	1.353	1.424	1.225	1.493	124.8	115.4
YEGJID01	1.352	1.418	1.237	1.492	126.3	114.5

References: CIBPIM: Smith *et al.* (1983 ▸); CIBPIM01: Bocelli *et al.* (1989 ▸); LASHEU: Panini *et al.* (2012 ▸); MANDIP: Goswami *et al.* (2005 ▸); MANDIP01: Fellowes (2020 ▸); MANDIP02: Romito & Bonifazi (2023 ▸); MANDIP03: Clarke *et al.* (2024 ▸); NUKVOH: McKay *et al.* (2020 ▸); YEGJID: Azumaya *et al.* (1994 ▸); YEGJID01: Gowda *et al.* (2008 ▸).

**Table 3:** Experimental details

Crystal data	
Chemical formula	C_19_H_15_NO
*M*_r_	273.32
Crystal system, space group	Triclinic, *P* 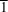
Temperature (K)	100
*a*, *b*, *c* (Å)	5.2935 (1), 12.0493 (2), 12.3713 (3)
α, β, γ (°)	65.411 (2), 80.417 (2), 80.644 (1)
*V* (Å^3^)	703.67 (3)
*Z*	2
Radiation type	Cu *K*α
μ (mm^−1^)	0.62
Crystal size (mm)	0.18 × 0.06 × 0.04

Data collection	
Diffractometer	XtaLAB Synergy, Dualflex, HyPix
Absorption correction	Multi-scan (*CrysAlis PRO*; Rigaku OD, 2021 ▸)
*T*_min_, *T*_max_	0.84, 1.0
No. of measured, independent and observed [*I* > 2σ(*I*)] reflections	20896, 2252, 2084
*R*_int_	0.030
(sin θ/λ)_max_ (Å^−1^)	0.581

Refinement	
*R*[*F*^2^ > 2σ(*F*^2^)], *wR*(*F*^2^), *S*	0.031, 0.081, 1.05
No. of reflections	2252
No. of parameters	194
H-atom treatment	H atoms treated by a mixture of independent and constrained refinement
Δρ_max_, Δρ_min_ (e Å^−3^)	0.11, −0.20

Computer programs: *CrysAlis PRO* (Rigaku OD, 2021 ▸), *SHELXT* (Sheldrick, 2015*a* ▸), *SHELXL* (Sheldrick, 2015*b* ▸), *ORTEPIII* (Burnett & Johnson, 1996 ▸); *ORTEP-3 for Windows* and *WinGX* (Farrugia, 2012 ▸) and *Mercury* (Macrae *et al.*, 2020 ▸).

## full crystallographic data

### N-Phenyl-[1,1'-biphenyl]-2-carboxamide Crystal data

**Table:**

C_19_H_15_NO	*Z* = 2
*M**_r_* = 273.32	*F*(000) = 288
Triclinic, *P*1	*D*_x_ = 1.290 Mg m^−^^3^
*a* = 5.2935 (1) Å	Cu *K*α radiation, λ = 1.54184 Å
*b* = 12.0493 (2) Å	Cell parameters from 15231 reflections
*c* = 12.3713 (3) Å	θ = 4.0–63.5°
α = 65.411 (2)°	µ = 0.62 mm^−^^1^
β = 80.417 (2)°	*T* = 100 K
γ = 80.644 (1)°	Needle, colorless
*V* = 703.67 (3) Å^3^	0.18 × 0.06 × 0.04 mm

### N-Phenyl-[1,1'-biphenyl]-2-carboxamide Data collection

**Table:**

XtaLAB Synergy, Dualflex, HyPix diffractometer	2252 independent reflections
Radiation source: micro-focus sealed X-ray tube, PhotonJet (Cu) X-ray Source	2084 reflections with *I* > 2σ(*I*)
Mirror monochromator	*R*_int_ = 0.030
Detector resolution: 10.0000 pixels mm^-1^	θ_max_ = 63.7°, θ_min_ = 4.0°
ω scans	*h* = −6→6
Absorption correction: multi-scan (CrysAlisPro; Rigaku OD, 2021)	*k* = −13→13
*T*_min_ = 0.84, *T*_max_ = 1.0	*l* = −14→13
20896 measured reflections	

### N-Phenyl-[1,1'-biphenyl]-2-carboxamide Refinement

**Table:**

Refinement on *F*^2^	Primary atom site location: structure-invariant direct methods
Least-squares matrix: full	Secondary atom site location: difference Fourier map
*R*[*F*^2^ > 2σ(*F*^2^)] = 0.031	Hydrogen site location: mixed
*wR*(*F*^2^) = 0.081	H atoms treated by a mixture of independent and constrained refinement
*S* = 1.05	*w* = 1/[σ^2^(*F*_o_^2^) + (0.0444*P*)^2^ + 0.1624*P*] where *P* = (*F*_o_^2^ + 2*F*_c_^2^)/3
2252 reflections	(Δ/σ)_max_ < 0.001
194 parameters	Δρ_max_ = 0.11 e Å^−^^3^
0 restraints	Δρ_min_ = −0.20 e Å^−^^3^

### N-Phenyl-[1,1'-biphenyl]-2-carboxamide Special details

**Table:**

Geometry. All esds (except the esd in the dihedral angle between two l.s. planes) are estimated using the full covariance matrix. The cell esds are taken into account individually in the estimation of esds in distances, angles and torsion angles; correlations between esds in cell parameters are only used when they are defined by crystal symmetry. An approximate (isotropic) treatment of cell esds is used for estimating esds involving l.s. planes.
Refinement. Hydrogen atoms bonded to carbon atoms were placed at geometrically idealized positions and refined using a riding model, with Uiso(H) = 1.2Ueq(C). The amide H atom was located in a difference-Fourier map and freely refined.

### N-Phenyl-[1,1'-biphenyl]-2-carboxamide Fractional atomic coordinates and isotropic or equivalent isotropic displacement parameters (Å^2^)

**Table:**

	*x*	*y*	*z*	*U*_iso_*/*U*_eq_	
O1	0.56138 (14)	0.48552 (7)	0.15047 (7)	0.0233 (2)	
N1	0.12096 (18)	0.51216 (8)	0.16680 (8)	0.0204 (2)	
H1	−0.016 (3)	0.4735 (12)	0.1824 (11)	0.026 (3)*	
C1	0.3539 (2)	0.44452 (10)	0.16721 (9)	0.0191 (3)	
C2	0.0753 (2)	0.63423 (10)	0.15990 (10)	0.0198 (3)	
C3	0.2518 (2)	0.72007 (10)	0.10018 (10)	0.0227 (3)	
H3	0.411682	0.698081	0.062454	0.027*	
C4	0.1921 (2)	0.83811 (10)	0.09629 (11)	0.0246 (3)	
H4	0.312790	0.896678	0.055944	0.029*	
C5	−0.0406 (2)	0.87187 (10)	0.15028 (10)	0.0243 (3)	
H5	−0.079582	0.952895	0.147088	0.029*	
C6	−0.2159 (2)	0.78608 (10)	0.20900 (10)	0.0239 (3)	
H6	−0.376344	0.808549	0.245972	0.029*	
C7	−0.1588 (2)	0.66771 (10)	0.21413 (10)	0.0220 (3)	
H7	−0.279691	0.609315	0.254787	0.026*	
C11	0.33708 (19)	0.31320 (10)	0.19073 (10)	0.0194 (3)	
C12	0.2034 (2)	0.28790 (10)	0.11718 (10)	0.0222 (3)	
H12	0.112167	0.353274	0.058094	0.027*	
C13	0.2018 (2)	0.16830 (10)	0.12918 (11)	0.0246 (3)	
H13	0.112241	0.151757	0.077916	0.029*	
C14	0.3319 (2)	0.07337 (10)	0.21654 (11)	0.0253 (3)	
H14	0.333382	−0.008692	0.224702	0.030*	
C15	0.4600 (2)	0.09738 (10)	0.29225 (11)	0.0239 (3)	
H15	0.545952	0.030985	0.352639	0.029*	
C16	0.4657 (2)	0.21702 (10)	0.28175 (10)	0.0204 (3)	
C21	0.5881 (2)	0.23759 (10)	0.37111 (10)	0.0210 (3)	
C22	0.8175 (2)	0.16885 (10)	0.41265 (10)	0.0251 (3)	
H22	0.901319	0.111695	0.380268	0.030*	
C23	0.9242 (2)	0.18298 (11)	0.50042 (11)	0.0303 (3)	
H23	1.079857	0.135190	0.527985	0.036*	
C24	0.8061 (2)	0.26608 (12)	0.54823 (11)	0.0327 (3)	
H24	0.880431	0.276035	0.608025	0.039*	
C25	0.5781 (2)	0.33477 (11)	0.50809 (11)	0.0296 (3)	
H25	0.495631	0.391962	0.540670	0.035*	
C26	0.4698 (2)	0.32056 (10)	0.42091 (10)	0.0239 (3)	
H26	0.312917	0.367838	0.394535	0.029*	

### N-Phenyl-[1,1'-biphenyl]-2-carboxamide Atomic displacement parameters (Å^2^)

**Table:**

	*U*^11^	*U*^22^	*U*^33^	*U*^12^	*U*^13^	*U*^23^
O1	0.0196 (4)	0.0220 (4)	0.0287 (5)	−0.0038 (3)	−0.0038 (3)	−0.0094 (3)
N1	0.0181 (5)	0.0181 (5)	0.0260 (5)	−0.0042 (4)	−0.0032 (4)	−0.0088 (4)
C1	0.0205 (6)	0.0205 (5)	0.0167 (6)	−0.0028 (4)	−0.0033 (4)	−0.0071 (4)
C2	0.0219 (6)	0.0187 (5)	0.0207 (6)	−0.0005 (4)	−0.0086 (4)	−0.0081 (5)
C3	0.0204 (6)	0.0222 (6)	0.0248 (6)	−0.0020 (4)	−0.0048 (5)	−0.0079 (5)
C4	0.0259 (6)	0.0203 (6)	0.0268 (6)	−0.0056 (5)	−0.0081 (5)	−0.0056 (5)
C5	0.0290 (6)	0.0194 (6)	0.0273 (6)	0.0005 (5)	−0.0114 (5)	−0.0101 (5)
C6	0.0232 (6)	0.0256 (6)	0.0257 (6)	0.0007 (5)	−0.0059 (5)	−0.0129 (5)
C7	0.0215 (6)	0.0228 (6)	0.0231 (6)	−0.0041 (4)	−0.0045 (5)	−0.0091 (5)
C11	0.0170 (5)	0.0205 (6)	0.0208 (6)	−0.0030 (4)	0.0011 (4)	−0.0090 (5)
C12	0.0222 (6)	0.0228 (6)	0.0212 (6)	−0.0032 (4)	−0.0028 (5)	−0.0081 (5)
C13	0.0264 (6)	0.0266 (6)	0.0258 (6)	−0.0062 (5)	−0.0023 (5)	−0.0143 (5)
C14	0.0266 (6)	0.0207 (6)	0.0314 (7)	−0.0029 (5)	−0.0010 (5)	−0.0139 (5)
C15	0.0217 (6)	0.0205 (6)	0.0278 (6)	0.0001 (4)	−0.0034 (5)	−0.0086 (5)
C16	0.0160 (5)	0.0224 (6)	0.0228 (6)	−0.0023 (4)	0.0006 (4)	−0.0099 (5)
C21	0.0206 (5)	0.0202 (5)	0.0201 (6)	−0.0059 (4)	−0.0010 (4)	−0.0052 (4)
C22	0.0214 (6)	0.0245 (6)	0.0255 (6)	−0.0051 (5)	−0.0026 (5)	−0.0053 (5)
C23	0.0250 (6)	0.0325 (7)	0.0271 (7)	−0.0087 (5)	−0.0077 (5)	−0.0020 (5)
C24	0.0379 (7)	0.0376 (7)	0.0235 (7)	−0.0158 (6)	−0.0076 (5)	−0.0072 (6)
C25	0.0377 (7)	0.0301 (6)	0.0229 (6)	−0.0112 (5)	−0.0011 (5)	−0.0107 (5)
C26	0.0242 (6)	0.0239 (6)	0.0228 (6)	−0.0048 (5)	−0.0019 (5)	−0.0078 (5)

### N-Phenyl-[1,1'-biphenyl]-2-carboxamide Geometric parameters (Å, º)

**Table:**

O1—C1	1.2283 (13)	C12—C13	1.3872 (16)
N1—C1	1.3626 (14)	C13—C14	1.3833 (17)
N1—C2	1.4198 (14)	C14—C15	1.3864 (16)
C1—C11	1.5001 (15)	C15—C16	1.3975 (15)
C2—C7	1.3905 (16)	C16—C21	1.4906 (16)
C2—C3	1.3920 (16)	C21—C22	1.3969 (15)
C3—C4	1.3882 (16)	C21—C26	1.3970 (16)
C4—C5	1.3851 (17)	C22—C23	1.3844 (17)
C5—C6	1.3857 (16)	C23—C24	1.3820 (19)
C6—C7	1.3859 (15)	C24—C25	1.3862 (18)
C11—C12	1.3919 (16)	C25—C26	1.3828 (17)
C11—C16	1.4098 (16)		
			
C1—N1—C2	126.70 (9)	C13—C12—C11	120.74 (10)
O1—C1—N1	123.91 (10)	C14—C13—C12	119.33 (11)
O1—C1—C11	122.02 (9)	C13—C14—C15	120.33 (10)
N1—C1—C11	114.07 (9)	C14—C15—C16	121.51 (10)
C7—C2—C3	119.80 (10)	C15—C16—C11	117.62 (10)
C7—C2—N1	117.46 (10)	C15—C16—C21	119.56 (10)
C3—C2—N1	122.73 (10)	C11—C16—C21	122.66 (10)
C4—C3—C2	119.36 (10)	C22—C21—C26	118.06 (10)
C5—C4—C3	121.06 (10)	C22—C21—C16	120.66 (10)
C4—C5—C6	119.23 (10)	C26—C21—C16	121.17 (10)
C5—C6—C7	120.42 (11)	C23—C22—C21	120.80 (11)
C6—C7—C2	120.13 (10)	C24—C23—C22	120.51 (11)
C12—C11—C16	120.43 (10)	C23—C24—C25	119.35 (11)
C12—C11—C1	118.88 (9)	C26—C25—C24	120.40 (12)
C16—C11—C1	120.61 (10)	C25—C26—C21	120.88 (11)

### N-Phenyl-[1,1'-biphenyl]-2-carboxamide Hydrogen-bond geometry (Å, º)

*Cg*1 is the centroid of the C2–C7 ring.

**Table:**

*D*—H···*A*	*D*—H	H···*A*	*D*···*A*	*D*—H···*A*
C3—H3···O1	0.95	2.38	2.9035 (15)	114
C7—H7···O1^i^	0.95	2.64	3.2433 (13)	122
N1—H1···O1^i^	0.877 (16)	2.307 (16)	3.0790 (12)	146.9 (13)
C13—H13···*Cg*1^ii^	0.95	2.78	3.5815 (13)	142
C24—H24···*Cg*1^iii^	0.95	2.94	3.8444 (13)	160

Symmetry codes: (i) *x*−1, *y*, *z*; (ii) −*x*, −*y*+1, −*z*; (iii) −*x*+1, −*y*+1, −*z*+1.
